# Inhibition of Notch Signaling Promotes the Differentiation of Epicardial Progenitor Cells into Adipocytes

**DOI:** 10.1155/2021/8859071

**Published:** 2021-04-09

**Authors:** Bin Liu, Dinghui Wang, Tianhua Xiong, Yajie Liu, Xiaodong Jing, Jianlin Du, Qiang She

**Affiliations:** Department of Cardiology, The Second Affiliated Hospital of Chongqing Medical University, Chongqing 400010, China

## Abstract

**Background:**

The role of Notch signaling pathway in the differentiation of epicardial progenitor cells (EPCs) into adipocytes is unclear. The objective is to investigate the effects of Notch signaling on the differentiation of EPCs into adipocytes.

**Methods:**

Frozen sections of *C57BL/6*J mouse hearts were used to observe epicardial adipose tissue (EAT), and genetic lineage methods were used to trace EPCs. EPCs were cultured in adipogenic induction medium with Notch ligand jagged-1 or *γ*-secretase inhibitor DAPT. The adipocyte markers, Notch signaling, and adipogenesis transcription factors were determined.

**Results:**

There was EAT located at the atrial–ventricular groove in mouse. By using genetic lineage tracing methods, we found that EPCs were a source of epicardial adipocytes. EPCs had lipid droplet accumulation, and the expression of adipocyte markers FABP-4 and perilipin-1 was upregulated under adipogenic induction. Activating the Notch signaling with jagged-1 attenuated the adipogenic differentiation of EPCs and downregulated the key adipogenesis transcription factor peroxisome proliferator activated receptor-*γ* (PPAR-*γ*), while inhibiting the signaling promoted adipogenic differentiation and upregulated PPAR-*γ*. When blocking PPAR-*γ*, the role of Notch signaling in promoting adipogenic differentiation was inhibited.

**Conclusions:**

EPCs are a source of epicardial adipocytes. Downregulation of the Notch signaling pathway promotes the differentiation of EPCs into adipocytes via PPAR-*γ*.

## 1. Introduction

Epicardial adipose tissue (EAT), located between the myocardium and epicardium, covers more than three-quarters of the human heart's surface and constitutes 20% of the mass of the human heart [1]. EAT acts as a buffer to protect the coronary arteries from torsion caused by arterial pulse waves and provides energy to the adjacent myocardium during energy limitation [1]. EAT contains not only adipocytes but also stromal cells and immune cells. It secretes numerous cytokines (including adipokines, interleukin, and chemokines) and is believed to be an active endocrine organ [2]. The associations between EAT amount and coronary artery disease, atrial fibrillation, sleep apnea syndrome, and metabolic syndrome have been demonstrated [3, 4]. Though EAT plays an important protective role and has pathophysiological significance, very little is known about its origin and expansion.

Epicardium is the outmost cell layer covering heart surface. In the developing heart, epicardial progenitor cells (EPCs) migrate to the outer surface of the heart to form the epicardium [5]. EPCs specifically express the T-box transcription factor (Tbx18) and Wilms tumor gene 1 (Wt1) in mouse heart. They influence the development of myocardium through secretion of growth factors and cytokines during embryonic stage. In addition, EPCs undergo epithelial-to-mesenchymal transition (EMT) and migrate into the myocardium to differentiate into smooth muscle cells and fibroblasts, which may also have the potential to differentiate into endothelial cells and cardiomyocytes [6]. Recently, several studies reported that EPCs have the capacity to differentiate into adipocytes [7–11]. The transcription factor peroxisome proliferator activated receptor-*γ* (PPAR-*γ*), insulin-like growth factor 1 receptor (IGF1-R), prokineticin-2, and atrial natriuretic peptide (ANP) regulate the adipogenic differentiation of EPCs [7, 8, 10, 11].

Notch signaling pathway plays an important role in regulating the differentiation and proliferation of EPCs [12, 13]. Four Notch receptors (Notch-1 to 4) and five ligands (Jagged-1, Jagged-2, Dll1, Dll3, and Dll4) are involved in the signal transduction process. Once the ligand binds to the Notch receptor, it is cleaved by *γ*-secretase, and then, the Notch intracellular domain (NICD) is formed [6]. Next, NICD enters the nucleus, combines with recombination signal binding protein J (RBP-j), and activates transcription of various target genes including Hes-1, Hey-1, and Hey-L. DAPT (N-[N-(3,5-Difluorophenacetyl-L-alanyl)]-S-phenylglycine-Butyl Ester), a *γ*-secretase inhibitor, prevents the cleavage of the Notch receptor and blocks the release of NICD. Jagged-1 and DAPT regulate the Notch signaling pathway and indirectly decide the fate of stem cells [14]. There is evidence showing that Notch signaling regulates the adipogenic differentiation of stem cells [15–17]. However, the role of Notch signaling in the differentiation of EPCs into adipocytes is not clear. Therefore, we investigated the effects of Notch signaling pathway on the adipogenic differentiation of EPCs in this study.

## 2. Methods

### 2.1. Mice

Animal experiments were in accord with the NIH Guide for the Care and Use of Laboratory Animals published and performed under protocols approved by the Animal Research Committee of Chongqing Medical University. Male and female *C57BL/6*J mice were purchased from the Laboratory Animal Center of the Chongqing Medical University and maintained under individually ventilated cages (IVC) condition. *Tbx18: Cre* knock-in mouse and *R26R^EYFP^*: the Cre lineage reporter mouse were introduced from Evans laboratory and Jackson Laboratory, respectively, in January, 2010. Both the mouse lines were based on the *C57BL/6*J mice background. We crossed the *Tbx18^Cre^* mouse with the *R26R^EYFP^* mouse and obtained *Tbx18^Cre^/R26R^EYFP^* double heterozygous mouse [18]. Tbx18 is specifically expressed in EPCs in mouse hearts, which can be used to trace the fate of EPCs in *Tbx18^Cre^/R26R^EYFP^* double heterozygous mouse.

### 2.2. Isolation and Culture of Mouse Epicardial Progenitor Cells

Isolation and culture of primary *C57BL/6*J mouse epicardial progenitors cells (EPCs) were described previously [6, 19]. Embryonic ventricular tissue was dissected from embryonic (E) day 11.5 mouse. Then, the ventricular tissue was placed in 12-well culture dishes coated with 1% gelatin and covered with sterile coverslips. The ventricular tissue was cultured at 37°C with 5% CO_2_ in a medium containing Dulbecco's modified Eagle medium (DMEM) supplemented with 10% fetal bovine serum (FBS) and 1% penicillin streptomycin. EPCs would migrate and expand from the edge of ventricular tissue after 24 hours. Then, the ventricular tissue was removed, and EPCs continued growing.

### 2.3. Adipogenic Induction

EPCs were maintained in DMEM with 10% fetal bovine serum (FBS) and 1% penicillin and streptomycin. To induce adipogenic differentiation, cells were cultured in adipogenic induction medium containing 2 *μ*M dexamethasone (Sigma, Cat: D1756), 0.5 mM 3-isobutyl-1-methylxanthine (IBMX; Sigma, Cat: I5879), and 10 *μ*g/ml insulin (Wanbang Biochemical Medicine, Cat: U-40) for at most 21 days. The medium was changed every 3 days.

### 2.4. Jagged-1, DAPT, and PPAR-*γ* Antagonist Treatment, Respectively

The Notch ligand jagged-1 peptide was purchased from R&D (R&D Systems, Minnesota, USA; Cat: 599-JG-100). Jagged-1 peptide was reconstituted at the concentration of 200 *μ*g/ml in sterile PBS following the manufacturer's instructions. To activate Notch signaling, jagged-1 peptide was added to the medium with the concentration of 100, 200, 500, and 1000 ng/ml. N-[N-(3,5-Difluorophenacetyl-L-alanyl)]-S-phenylglycine-Butyl Ester (DAPT) was purchased from MCE (MedChemExpress, Cat: HY-13027). DAPT was prepared in dimethyl sulfoxide (DMSO) and stored at -20°C. To inhibit Notch signaling, DAPT was added to the medium with the concentration of 1, 2, 5, and 10 *μ*M. GW9662 (MCE, NJ, USA; Cat: HY-16578) was an effective and selective PPAR-*γ* antagonist, and the concentration of 10 *μ*M of GW9662 was used to inhibit PPAR-*γ* [20]. The EPCs treated with DMSO served as control.

### 2.5. RNA Isolation and Real-Time Quantitative Polymerase Chain Reaction (RT-PCR)

Total RNA was extracted from about 1∗10^7^ cells with Trizol reagent (Takara, Cat: 9108) according to the manufacturer's instructions. For reverse transcription PCR, RNA was converted into cDNA using the Prime Script Reverse Transcriptase Kit (Takara, Cat: RR047A). The reverse transcription was performed as follows: 37°C for 15 s and 85°C for 5 s, followed by 4°C for 4 mins. Quantitative real-time PCR (qRT-PCR) was performed on a C1000 thermal cycler (BioRad) under the following conditions: 95°C for 30s followed by 95°C for 5 s, 60°C for 30 s, and 95°C for 30 s for 39 cycles. The mRNA expression levels of each sample were calculated using the comparative method (*ΔΔ*CT) with GAPDH as the endogenous control [21].

### 2.6. Immunofluorescence

Cells were fixed in PBS with 4% PFA for 20 mins, washed with PBS, and incubated with 10% goat serum for blocking nonspecific binding. The cells were subsequently stained with the following antibodies: rabbit anti-Tbx18 (Abcam, Cat: ab115262), rabbit anti-Wt1 (Abcam, Cat: ab180840), rabbit anti-perilipin-1 (Abcam, Cat: ab3526), rabbit anti-FABP4 (fatty acid binding protein 4; Abcam, Cat: ab92501), rabbit anti-activated Notch-1 (Abcam, Cat: ab52301), mouse anti-Hes-1(Santa, Cat: sc-166410), rabbit anti-C/EBP-*α* (Cell signaling Technology, Cat: 8178T), rabbit anti-PPAR-*γ* (Cell signaling Technology, Cat: 2435T), rabbit anti-*α*-SMA (Abcam, Cat: ab124964), rabbit anti-Myh11 (Abcam, Cat: ab224804), and mouse anti-Periostin (Santa, Cat: sc-398631) at 4°C for at least 12 hours [22]. Then, the cells were incubated with Cy3-conjugated goat anti-rabbit IgG (Proteintech, Cat: SA00009-2), Alexa Fluor 647-conjugated goat anti-rabbit IgG (Abcam, Cat: ab150079), or FITC-conjugated goat anti-mouse IgG (Proteintech, Cat: SA00003-1) at 37°C for 30 mins, and nucleus was stained with 4′, 6-diamidino-2-phenylindole (DAPI, Beyotime, Cat: C1005) at room temperature for 6 mins. For frozen slice, mouse hearts were isolated, fixed in PBS with 4% PFA for 24 hours, dehydrated in 15% sucrose solution for 12 hours and 30% for 6 hours, embedded in optimal cutting temperature compound (OCT), and sectioned into 10 *μ*m thick sections. The sections were washed with PBS, incubated with 10% goat serum for blocking nonspecific binding, and stained with the following antibodies: rabbit anti-perilipin-1 (Abcam, Cat: ab3526), rabbit anti-FABP-4 (Abcam, Cat: ab92501), rabbit anti-activated Notch-1 (Notch-1 intracellular domain, NICD) (Abcam, Cat: ab52301), rabbit anti-Notch-1 (Cell signaling Technology, Cat: 4380T), and rabbit anti-Jagged-1(Cell signaling Technology, Cat: 70109T) at 4°C for at least 12 hours. Then, the slices were incubated with Cy3-conjugated goat anti-rabbit IgG (Proteintech, Cat: SA00009-2) and Alexa Fluor 647-conjugated goat anti-rabbit IgG (Abcam, Cat: ab150079) at 37°C for 30 mins, and nucleus was stained with 4′, 6-diamidino-2-phenylindole (DAPI) at room temperature for 6 mins.

### 2.7. Oil Red O Staining

Cells and frozen tissue sections were fixed in PBS with 4% PFA for 20 mins at room temperature, washed with PBS three times for 5 mins each time, and infiltrated with 60% isopropanol for 3 mins. The cells and tissue sections were then stained with Oil Red O (0.3% in 60% isopropanol) at room temperature for 30 mins and washed with 60% isopropanol for 30 s, stained with hematoxylin for 20 s, and washed with tap water for 5 mins.

### 2.8. Hematoxylin and Eosin Staining

Since adipose tissue will be dissolved by organic solvents, the tissue cannot be embedded in paraffin. Therefore, frozen slices were used for HE staining. The slices were stained with hematoxylin stain for 30 s and washed with tap water for 5 mins. Later, they were stained with eosin for 10 s and washed with tap water for 10 mins.

### 2.9. Statistical Analysis

The statistical analyses were performed using the SPSS Version 20.0 software. The data were expressed as the means ± SD. The differences between two groups were calculated by *t*-test. Differences between more than two groups were assessed by One-way ANOVA. *p* values <0.05 were considered statistically significant.

## 3. Results

### 3.1. Mice Have EAT, and Tbx18^+^ EPCs Are a Source of Epicardial Adipocytes

**Figure 1:**
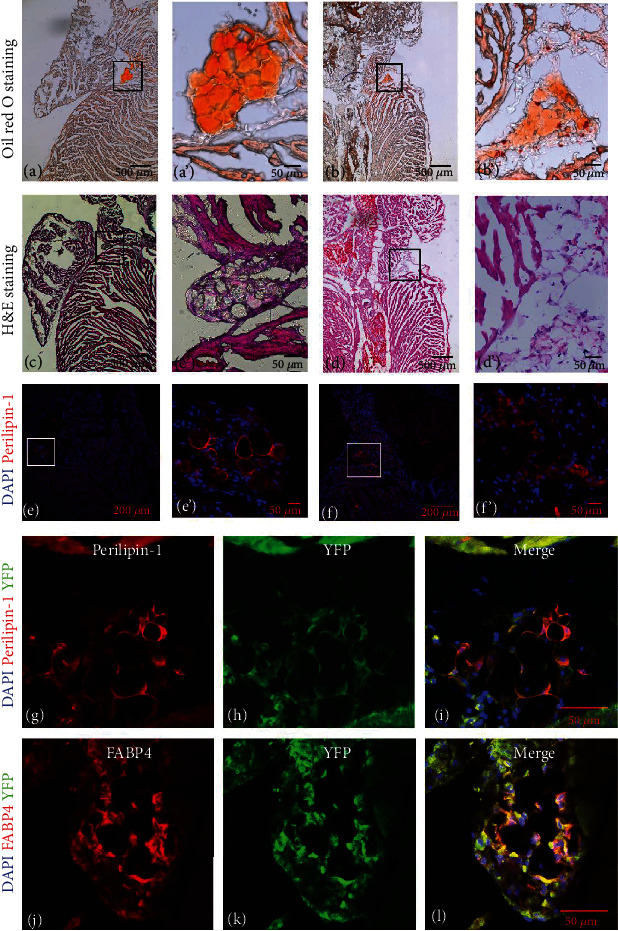
EAT located at the left and right atrial–ventricular groove in postnatal 8-week-old mouse hearts. (a, b) Oil red O staining showed that EAT located at the atrial–ventricular groove in mouse. The boxed regions were shown at higher magnification in A' and B'. (c, d) H&E staining showed that EAT was present in the atrial–ventricular groove underneath the epicardium. The boxed regions were shown at higher magnification in C' and D'. (e, f) Immunofluorescence staining for adipocyte marker perilipin-1 showed the epicardial adipocytes were present in the right and left atrial–ventricular groove. The boxed regions were shown at higher magnification in E' and F'. (g–l) The genetic lineage tracing methods were used to trace the EPCs. Immunofluorescence staining for adipocyte markers perilipin-1 (red) and FABP4 (red) was partly colocalized with the lineage marker EYFP (green) in the same section.

We observed the hearts of mouse at different ages to seek the epicardial adipose tissue (EAT), including embryonic 11.5 days (E11.5), neonatal, and postnatal 2-, 4-, and 8-week-old mice. However, we did not find EAT in E11.5, neonatal, and 2- and 4-week-old mouse with oil red O staining (supplement Fig. 1). Instead, we found EAT in 8-week-old mouse located at the left and right atrial–ventricular groove (Figures 1(a)–1(d)). We also found EAT by determining the adipocyte specific marker perilipin-1(Figures 1(e) and 1(f)). Tbx18 was specifically expressed in EPCs in mouse hearts, and EPCs were labeled as they were initially formed in the *Tbx18^Cre^/R26R^EYFP^* heterozygous mouse. We used the genetic lineage tracing methods to investigate the source of epicardial adipocytes. The results showed that some of the perilipin-1^+^ and FABP4^+^ adipocytes colocalized with the lineage marker EYFP (Figures 1(g)–1(l)), indicating that parts of the epicardial adipocytes were derived from EPCs.

### 3.2. Culture and Identification of EPCs from Embryonic Day 11.5 Mouse Hearts

**Figure 2:**
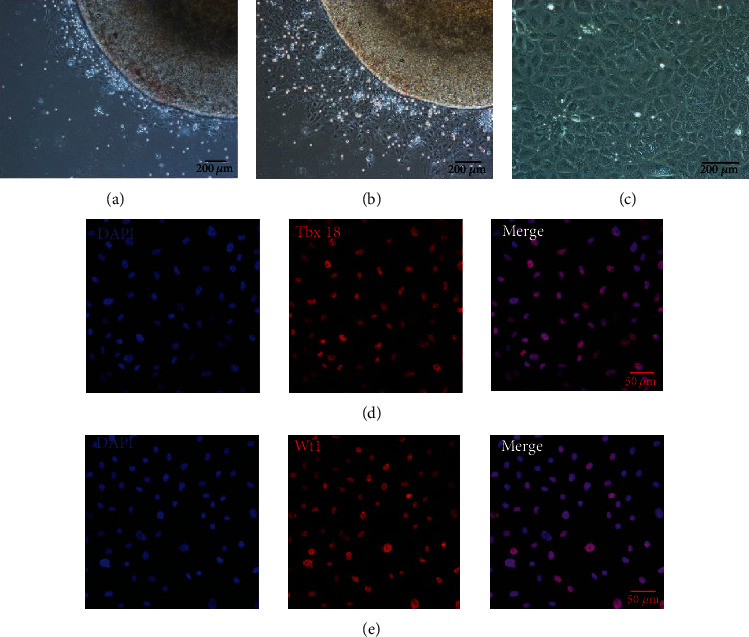
EPCs migrated out from the edge of embryonic mouse heart. (a) The cobblestone-like EPCs grew from the edge of the embryonic ventricular tissue after 24 hours' culture. (b) The cobblestone-like EPCs continued growing from the edge of the embryonic ventricular tissue after 48 hours' culture. (c) Cobblestone-like EPCs were observed after removing the ventricular tissue. (d, e) Immunofluorescence staining showed all cultured EPCs expressed Tbx18 and Wt1.

To explore the effect of Notch signaling on the adipogenic differentiation of EPCs, we cultured EPCs using embryonic day (E) 11.5 embryo mouse ventricular tissue. Cells could be seen to migrate out from the edges of the ventricular tissue after 24 hours' culture, showing cobblestone-like morphology and expanding outward around the tissue mass (Figure 2(a)). The cells continued growing and displayed a uniform epithelioid morphology (Figures 2(b) and 2(c)). To identify the primary cells property, EPCs' specific markers Wt1 and Tbx18 were determined by immunofluorescence staining. The results showed that all the cells expressed EPCs' specific markers Tbx18 and Wt1 (Figures 2(d) and 2(e)). To sum up, we confirmed that the cultured cells were EPCs according to their morphological and specific markers.

### 3.3. Jagged-1 Activates While DAPT Inhibits the Notch Signaling in EPCs

**Figure 3:**
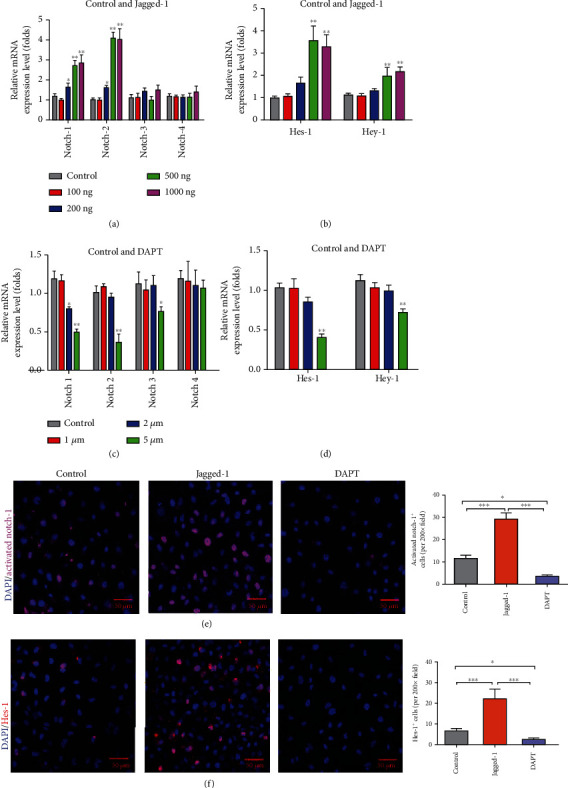
Notch signaling and its target genes in EPCs were regulated by the treatment of jagged-1 and DAPT for 2 days. (a, b) The mRNA expression of Notch signaling and its target genes were significantly upregulated with the treatment of jagged-1 for 2 days compared with the control. (c, d) The mRNA expression of Notch signaling and its target genes were significantly downregulated with the treatment of DAPT for 2 days compared to the control. The *p* values were calculated by *t*-test. ^∗^*p* < 0.05, ^∗∗^*p* < 0.01, and ^∗∗∗^*p* < 0.001 for pairwise comparison. (e) Immunofluorescence staining showed that activated Notch-1 was significantly upregulated with jagged-1 treatment while decreased with DAPT treatment. (f) Immunofluorescence staining showed that Hes-1 was significantly upregulated with jagged-1 treatment while decreased with DAPT treatment. The *p* values were calculated by ANOVA. ^∗^*p* < 0.05, ^∗∗^*p* < 0.01, and ^∗∗∗^*p* < 0.001 for pairwise comparison among the groups.

To determine the optimal concentrations of jagged-1 for activating Notch signaling and DAPT for inhibiting Notch signaling, EPCs were cultured in adipogenic induction medium with jagged-1 (100, 200, 500, and 1000 ng/ml) or DAPT (1, 2, 5, and 10 *μ*M) for 2 days. The Notch signaling related genes Notch-1 to -4 and the target genes Hes-1 and Hey-1 were determined. The results showed that jagged-1 treatment enhanced the mRNA expression levels of Notch-1, Notch-2, Hes-1, and Hey-1 with a maximum effect at 500 ng/ml jagged-1, but 1000 ng/ml jagged-1 did not further activate the Notch signaling (Figures 3(a) and 3(b)). In DAPT treatment group, the concentration of 10 *μ*M DAPT led to cells death, and 5 *μ*M DAPT significantly inhibited the Notch signaling and its target genes (Figures 3(c) and 3(d)). Consistent with the mRNA expression, the protein levels of activated Notch-1 and Hes-1 in EPCs were significantly upregulated with 500 ng/ml jagged-1 treatment while decreased in 5 *μ*M DAPT treatment (Figures 3(e) and 3(f)). These data indicated that 500 ng/ml jagged-1 activated Notch signaling while 5 *μ*M DAPT inhibited Notch signaling, which was used in subsequent experiments.

### 3.4. Inhibition of Notch Signaling Promotes the Adipogenic Differentiation of EPCs

**Figure 4:**
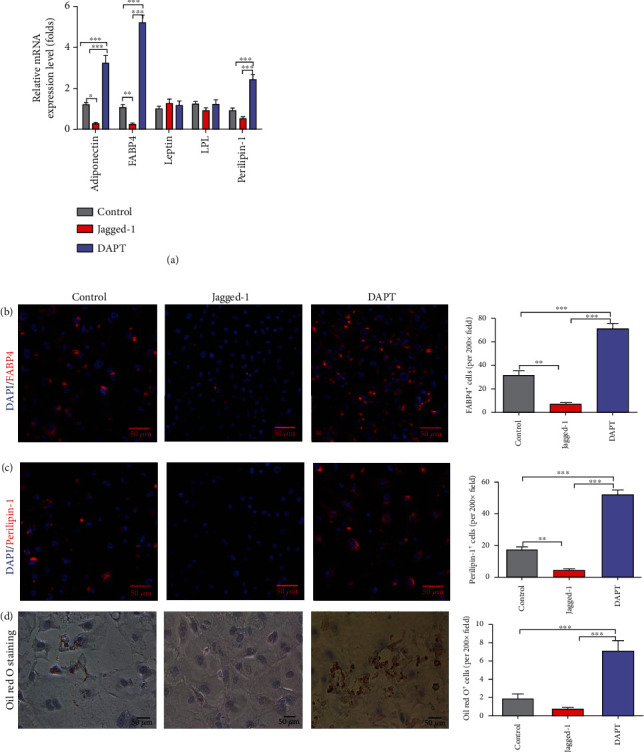
Inhibition of Notch signaling promoted the adipogenic differentiation of EPCs. (a) The mRNA expression levels of adipocyte-related genes adiponectin, FABP4, leptin, LPL, and perilipin-1 were determined by RT-PCR after EPCs cultured in adipogenic induction medium alone, with jagged-1, or with DAPT for 7 days. (b, c) The adipocyte markers FABP-4 and perilipin-1 were determined by immunofluorescence staining after EPCs were cultured in adipogenic induction medium alone, with jagged-1, or with DAPT for 7 days. (d) Lipid droplet accumulation was detected by Oil red O staining after EPCs cultured in adipogenic induction medium alone, with jagged-1, or with DAPT for 21 days. The *p* values were calculated by ANOVA. ^∗^*p* < 0.05, ^∗∗^*p* < 0.01, and ^∗∗∗^*p* < 0.001 for pairwise comparison among the groups.

To evaluate the effects of Notch signaling on adipogenesis of EPCs, we activated the Notch signaling with jagged-1 and inhibited the Notch signaling with DAPT under adipogenic induction. After 7 days' culture, adipocyte-related proteins were determined to assess the effects of Notch signaling on adipogenesis. Jagged-1 treatment significantly decreased the mRNA expression levels of adipogenic markers of adiponectin and fatty acid binding protein-4 (FABP-4), while DAPT treatment increased the mRNA expression of adiponectin, FABP4, and perilipin-1 (Figure 4(a)). To confirm the mRNA expression results, we determined the adipocyte markers FABP-4 and perilipin-1 by immunostaining. Consistent with the mRNA expression results, the proportions of FABP4 and perilipin-1 significantly decreased in the jagged-1 treatment group while increased in the DAPT treatment group (Figures 4(b) and 4(c)). However, Oil red O staining showed that there was no significant lipid accumulation in each group after 7- and 14-day culture. There was lipid accumulation after 21-day adipogenic induction culture, and lipid accumulation increased in the DAPT treatment group but decreased in the jagged-1 treatment group (Figure 4(d)). These results indicated that jagged-1 treatment attenuated adipogenic differentiation of EPCs, while DAPT treatment enhanced adipogenic differentiation.

### 3.5. Notch Signaling Is Gradually Downregulated in Mouse Epicardium

**Figure 5:**
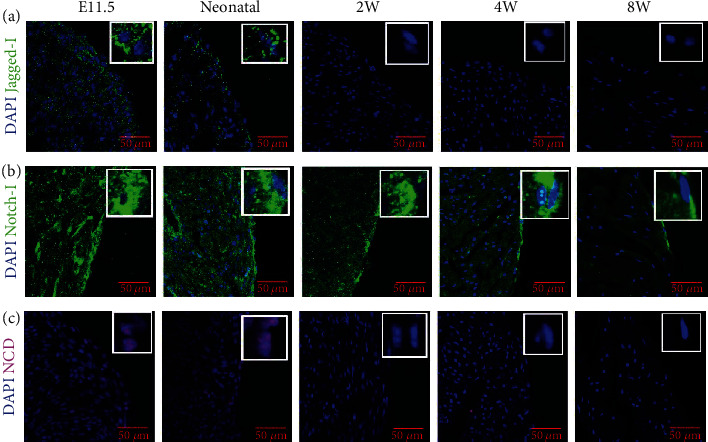
The dynamic changes of Notch signaling in mouse epicardium. (a) The dynamic changes of Jagged-1 in mouse epicardium at different ages. Jagged-1 was mainly expressed in the heart of E11.5 and neonatal mice. (b) The dynamic changes Notch-1 in mouse epicardium at different ages. Notch-1 expression in the mouse epicardium gradually decreased from E11.5 to postnatal 8-week-old mice. (c) The dynamic changes of NICD (activated Notch-1) in epicardium in mice at different ages. NICD was found in epicardium at E11.5 and neonatal mouse but not found in epicardium at postnatal 2-, 4-, and 8-week-old mice.

In order to observe the dynamic changes of Notch signaling in epicardial cells in mice, we determined Jagged-1, Notch-1, and NICD (Notch-1 intracellular domain (NICD)) in E11.5, neonatal, and postnatal 2-, 4-, and 8-week-old mouse hearts. We found that Jagged-1 was mainly expressed in the epicardium of E11.5 and neonatal mice but not obvious in 2-, 4-, and 8-week-old mice (Figure 5(a)). Notch-1 was continuously expressed in mouse epicardium at different ages, but the expression was the highest at E11.5 and neonatal (Figure 5(b)). NICD was only expressed in E11.5 and neonatal mice but not obvious in epicardium at postnatal 2-, 4-, and 8-week-old mice (Figure 5(c)), which was consistent with the expression of Jagged-1.

### 3.6. Notch Signaling Inhibits Adipogenic Differentiation of EPCs via PPAR-*γ*

**Figure 6:**
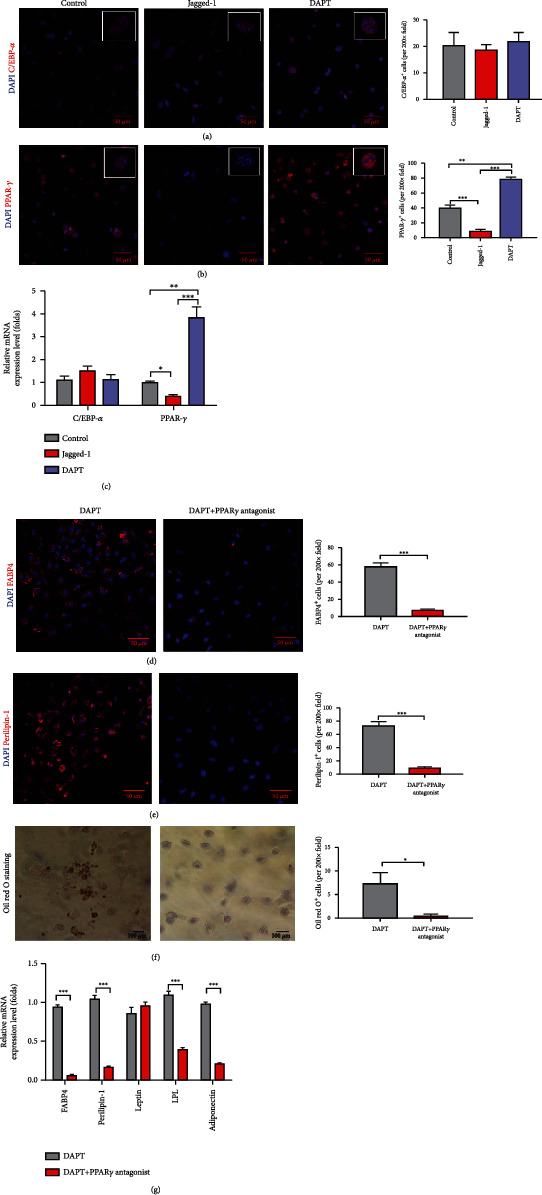
DAPT treatment upregulated the transcription factor PPAR-*γ*. (a, b) The crucial adipogenic transcription factors PPAR-*γ* and the C/EBP-*α* were determined by immunofluorescence staining after EPCs were cultured in adipogenic induction medium alone, with jagged-1 or with DAPT for 7 days. (c) The mRNA expression levels of PPAR-*γ* and C/EBP-*α* were determined by RT-PCR. (d, e) The adipocyte markers FABP-4 and perilipin-1 were determined by immunofluorescence. EPCs were cultured in adipogenic induction medium with DAPT alone or combined with PPAR-*γ* antagonists. (f) Lipid droplet accumulation was determined by Oil red O staining. EPCs were cultured in adipogenic induction medium with DAPT alone or combined with PPAR-*γ* antagonists. (g) The mRNA expression levels of adipocyte-related genes FABP4, perilipin-1, leptin, LPL, and adiponectin were determined by RT-PCR. The *p* values were calculated by *t*-test between 2 groups and by ANOVA among 3 groups. ^∗^*p* < 0.05, ^∗∗^*p* < 0.01, and ^∗∗∗^*p* < 0.001 for pairwise comparison among the groups.

To understand the underlying mechanisms of Notch signaling in adipogenesis in vitro, we determined the master transcription factor peroxisome proliferator activated receptor-*γ* (PPAR-*γ*) and CCAAT/enhancer binding protein-*α* (C/EBP-*α*) for adipogenesis. However, we did not find significant difference of C/EBP-*α* among the three groups (Figures 6(a) and 6(c)). Interestingly, we found that PPAR-*γ* increased with DAPT treatment while decreased with Jagged-1 treatment (Figures 6(b) and 6(c)). We speculated that Notch signaling inhibited the adipogenic differentiation of EPCs through the transcription factor PPAR-*γ*. Therefore, we used DAPT to downregulate Notch signaling and PPAR-*γ* antagonists to block the transcription role of PPAR-*γ*. We found that PPAR-*γ* antagonists significantly downregulated the expression of FABP4 and perilipin-1 and reduced the formation of lipid droplets (Figures 6(d)–6(f)). The mRNA expression of adipocyte-related genes was also downregulated (Figure 6(g)).

### 3.7. Inhibition of Notch Signaling Reduced the Differentiation of EPCs into Smooth Muscle Cells

**Figure 7:**
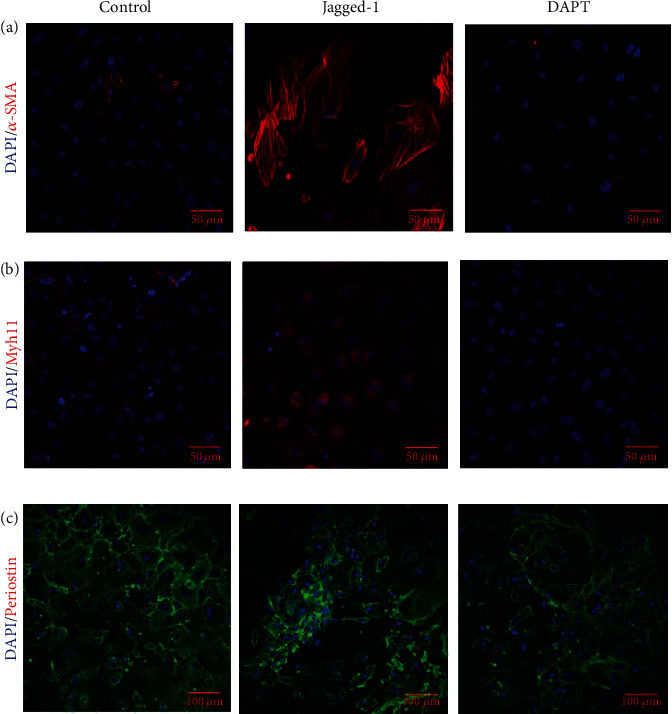
DAPT treatment reduced the differentiation of EPCs into smooth muscle cells. (a, b) Jagged-1 treatment increased the expression of smooth muscle cell specific marker *α*-SMA and Myh11, while DAPT reduced their expression. (c) Jagged-1 and DAPT treatment did not affect the expression of fibroblast specific marker periostin.

EPCs have the capacity to spontaneously differentiate into smooth muscle cells and fibroblasts in vitro. To determine the effect of Notch signaling on the smooth muscle cells and fibroblast differentiation, EPCs were cultured in the medium with Jagged-1 or DAPT for 7 days. We found that the smooth muscle cells specific markers *α*-smooth muscle actin (*α*-SMA) and myosin heavy chain 11 (Myh11) increased with Jagged-1 treatment while decreased with DAPT treatment (Figures 7(a) and 7(b)). However, we did not find significant differences of the fibroblast specific marker periostin among the groups (Figure 7(c)).

## 4. Discussion

In this study, we found epicardial adipose tissue (EAT) located at the atrial–ventricular groove in mice, and epicardial progenitor cells (EPCs) were a source of epicardial adipocytes. EPCs had the potential to differentiate into adipocytes under adipogenic induction in vitro. Upregulation of Notch signaling with jagged-1 attenuated the adipogenesis of EPCs, while inhibition of Notch signaling with DAPT enhanced the adipogenic differentiation. Simultaneously, Notch signaling acted as an upstream of PPAR-*γ* and inhibited the adipogenic differentiation of EPCs via PPAR-*γ*.

The earlier studies reported that there was no EAT in commonly used experimental animals including amphibians, avian, and rodents, but the latest studies showed that adult mice have EAT located at the atrial and atrial-ventricular groove [7, 8]. Our study also confirmed that mice have EAT located at the left and right atrial-ventricular groove, but we did not find EAT outside the atrial-ventricular groove. EAT was gradually formed after birth accompany by the Notch signaling downregulation. Epicardium is likely to be a source of epicardial adipocytes and contributes to the formation and expansion of EAT. Myocardial injury, high-fat diet, and ANP trigger the adipogenesis process of EPCs and promote the differentiation of EPCs into epicardial adipocytes [8, 9]. However, the ratio of epicardial adipocytes derived from EPCs remained controversial. Using the lineage tracing method, our study indicated that epicardial adipocytes were partly derived from EPCs.

Mouse EPCs have been demonstrated to be pluripotent, which can differentiate into smooth muscle cells and fibroblasts, even chondrocyte and osteoblast lineages, when cultured in different induction conditions [9]. It is reported that mouse epicardial cells can differentiate into epicardial adipocytes under the conditions of cardiac injury in vivo [9, 10]. PPAR-*γ*, IGF-1R, ANP, and prokineticin-2 have been demonstrated to be involved in the adipogenic differentiation of EPCs [7, 8, 10, 11]. The Notch signaling pathway has been shown to play an important role in cell proliferation and differentiation in stem cells [23]. Notch activation inhibits adipogenesis in primary human bone marrow stromal cells and even drives adipocyte dedifferentiation in mouse [16, 17]. Notch ligand Jagged-1 reduced the differentiation of 3T3-L1 preadipocytes into adipocytes by upregulating the Hes-1 expression. The *γ*-secretase inhibitor, DAPT, blocks the activation of Notch signaling and promotes the adipogenic differentiation of stem cells [24]. Up to now, the role of Notch signaling on adipogenesis of EPCs has not been investigated. In our study, we found that EPCs have the potential to differentiate into adipocytes under adipogenic induction in vitro. The adipogenic differentiation of EPCs was attenuated by upregulation of Notch signaling but enhanced by downregulation of Notch signaling. In addition, Notch signaling pathway was gradually downregulated in mouse hearts after birth, which was accompanied by the formation of EAT. It indicated that downregulation of Notch signaling may promote the differentiation of EPCs into epicardial adipocytes. We also found that activation of Notch signaling promoted the differentiation of EPCs into smooth muscle cells. These results showed that Notch signaling affected the fate of EPCs.

The transcription factor nuclear receptor PPAR-*γ* and C/EBP-*α* are believed to be crucial for conversion of stem cells to adipocytes, and C/EBP-*α* acts as the upstream regulator of PPAR-*γ* [25]. PPAR-*γ* has been shown to play an important role in adipocyte differentiation, which is regarded as an essential and sufficient factor to induce adipogenic differentiation [26, 27]. We determined the transcription factors PPAR-*γ* and C/EBP-*α* in the adipogenesis of EPCs. We found that both the expression of C/EBP-*α* and PPAR-*γ* were very low under adipogenic induction alone. That was why there were not many adipocytes formed under the adipogenic induction alone. In addition, we found that downregulation of the Notch signaling promoted adipogenic differentiation of EPCs and enhanced the PPAR-*γ* expression. When blocking PPAR-*γ* transcription, the adipogenic effects of downregulation of Notch signaling disappeared. These results indicated that Notch signaling regulated the adipogenic differentiation of EPCs via PPAR-*γ*. The Notch signaling pathway has been shown to act as an upstream for PPAR-*γ* [15]. Upregulation of Notch signaling inhibits the PPAR-*γ* expression and vice versa [28]. Hes-1 is a target gene of the Notch signaling. Previous studies indicate that upregulation of Hes-1 suppresses the expression of PPAR-*γ*, which prevents the adipogenic differentiation of mesenchymal stem cells [15]. In our study, we found that downregulation of Hes-1 was accompanied by the upregulation of the PPAR-*γ* expression. These indicated that the transcription of PPAR-*γ* may be regulated by the Notch-Hes-1 signaling pathway in EPCs. It has been shown that an increase in EAT contributes to the pathogenesis of coronary heart disease, atrial fibrillation, and heart failure by favoring fibrosis and inflammation [29, 30]. Reducing an increase in EAT may help the treatment of these diseases. Our study suggests that Notch signaling may be a target to reduce the expansion of EAT.

### 4.1. Limitations

This study has potential limitations. Firstly, we observed the dynamic changes of the Notch signaling pathway in the developing heart, but we did not down- or upregulate the Notch signaling to observe the effect on the adipogenic differentiation of EPC in vivo. Secondly, previous studies showed that myocardial infarction, myocardial frostbite, and heart failure induce the differentiation of EPCs into epicardial adipocytes [7–10], but we did not investigate the changes of Notch signaling pathway and its effects on adipogenic differentiation of EPCs under pathological conditions.

In summary, we confirm that mice have EAT (EAT) located at the atrial-ventricular groove. EPCs have the potential to differentiate into adipocytes in vivo and vitro. Downregulation of Notch signaling pathway promotes the adipogenic differentiation of EPCs via PPAR-*γ*.

## Data Availability

The datasets used and/or analyzed in the present study are available from the corresponding author on reasonable request.

## Supplementary Materials

Supplementary Materials Figure S1: Oil red O staining showed that there was no EAT in the left and right atrial–ventricular groove in E11.5, neonatal, postnatal 2- and 4-week-old mice. EAT: epicardial adipose tissue; E: embryonic.
