# Supplementary material for: Inhibition of Notch Signaling Promotes the Differentiation of Epicardial Progenitor Cells into Adipocytes
Source: Stem Cells Int. 2021 Apr 9;2021:8859071. doi: 10.1155/2021/8859071 (PMC8052169; doi:10.1155/2021/8859071)
Supplement: Supplementary Materials — Figure S1: Oil red O staining showed that there was no EAT in the left and right atrial–ventricular groove in E11.5, neonatal, postnatal 2- and 4-week-old mice. EAT: epicardial adipose tissue; E: embryonic. [file 8859071.f1.docx]

**Supplement information**


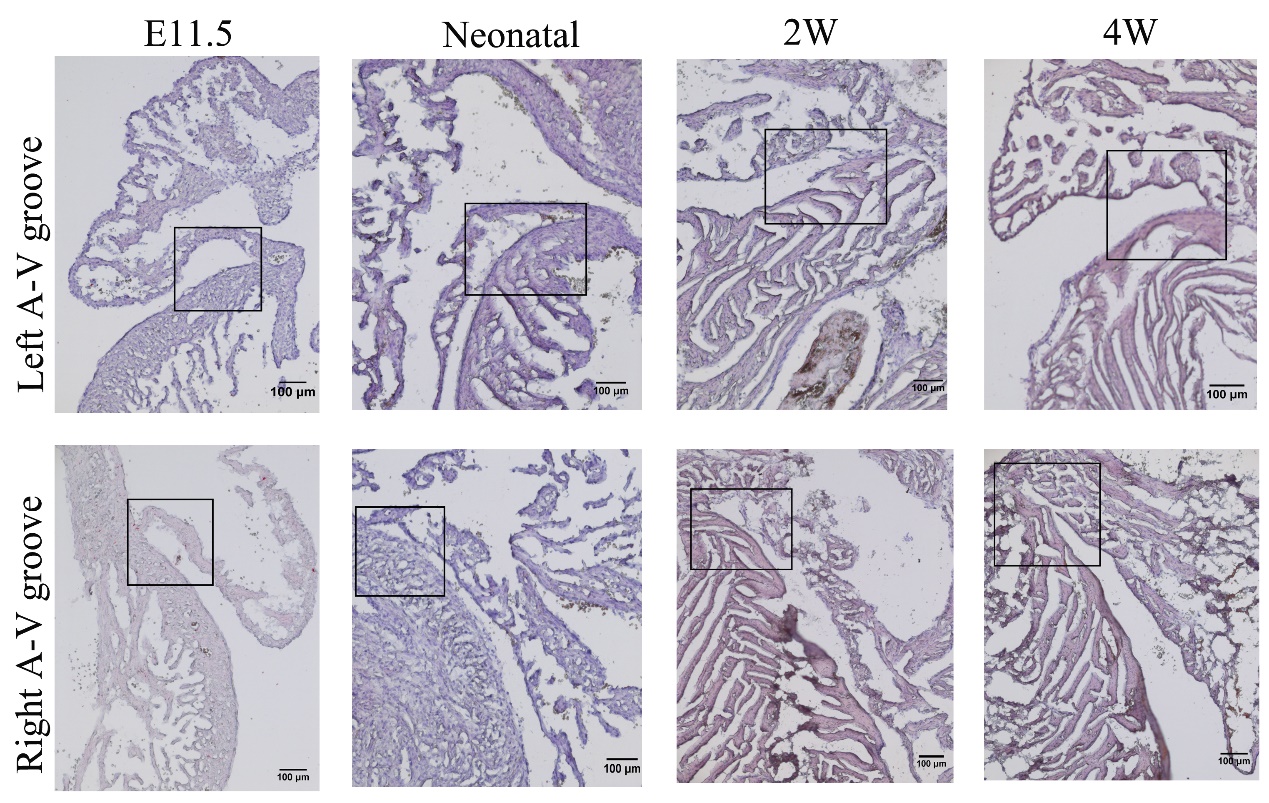


Fig. S1 Oil red O staining showed that there was no EAT in the left and right atrial–ventricular groove in E11.5, neonatal, postnatal 2- and 4-week-old mice. EAT: epicardial adipose tissue, E: embryonic.
